# Evaluation of oxidative stress, 3-Nitrotyrosine, and HMGB-1 levels in patients with wet type Age-Related Macular Degeneration

**DOI:** 10.5937/jomb0-32189

**Published:** 2022-07-29

**Authors:** Zor Kürşad Ramazan, İsmail Sarı, Biçer Gamze Yıldırım, İnayet Güntürk, Erkut Küçük, Serpil Erşan, Gönül Şeyda Seydel

**Affiliations:** 1 Niğde Ömer Halisdemir University School of Medicine Department of Ophthalmology, Bor Yolu, Niğde, Turkey; 2 Niğde Ömer Halisdemir University School of Medicine Department of Biochemistry, Bor Yolu, Niğde, Turkey; 3 Niğde Ömer Halisdemir University, Healthcare Services, Zübeyde Hanım Health Services Vocational High School, Bor Yolu, Niğde, Turkey; 4 Niğde Ömer Halisdemir University School of Medicine Department of Medical Biochemistry, Bor Yolu, Niğde, Turkey

**Keywords:** HMGB-1, 3-Nitrotyrosine, TAS, TOS, OSI, wet type AMD, HMBG-1, 3-nitrotirozin, TAS, TOS, OSI, mokri tip AMD

## Abstract

**Background:**

This study aims to compare serum HMGB-1, 3-nitrotyrosine (3-NT), TAS, TOS, and OSI levels in Wettype Age-Related Macular Degeneration (wAMD) patients and healthy controls to determine the correlation of these parameters with each other.

**Methods:**

Thirty patients with Wet-type Age-Related Macular Degeneration (wAMD) and 27 healthy adults, as controls were enrolled in the study. We determined the TAS and TOS levels in serum samples of both groups using commercial kits on a microplate reader. Serum HMGB-1 and 3-NT levels were measured with the enzyme-linked immunosorbent assay method.

**Results:**

HMGB-1 levels were significantly higher in the patient group (137.51 pg/mL, p=0.001), while there was no difference between the two groups in serum 3-NT levels (p=0.428). A statistically significant difference found in the levels of TOS and OSI (p=0.001 and p=0.045, respectively) between the patients and controls, however, no significant difference was observed between the groups in terms of TAS levels (p=0.228).

**Conclusions:**

Oxidative stress and HMGB-1 levels were increased in wAMD patients and enhanced oxidative stress may be associated with increased tissue necrosis and inflammation. Thus administration of antioxidant treatment in addition to routine therapy should be considered in wAMD.

## Abstract (sr)

**Uvod:**

Ova studija ima za cilj da uporedi serumske nivoe HMBG-1, 3-nitrotirozina (3-NT), TAS, TOS i OSI kod pacijenata sa vlažnom starosnom makularnom degeneracijom (vAMD) i zdravih kontrola kako bi se utvrdila korelacija ovih parametri međusobno.

**Metode:**

Trideset pacijenata sa vlažnom starosnom makularnom degeneracijom (wAMD) i 27 zdravih odraslih osoba su uključene u studiju. Određivali smo nivoe TAS i TOS u uzorcima seruma obe grupe pomoću komercijalnih kompleta na čitaču mikroploča. Serumski nivoi HMBG-1 i 3-NT mereni su pomoću enzimsko-imunološke analize.

**Rezultati:**

Nivoi HMBG-1 bili su značajno veći u grupi pacijenata (137,51 pg/mL, p = 0,001), dok nije bilo razlike između dve grupe u serumskim nivoima 3-NT (p = 0,428). Statistički značajna razlika pronađena u nivoima TOS i OSI (p = 0,001 i p = 0,045, respektivno) između pacijenata i kontrola, međutim, nije primećena značajna razlika između grupa u pogledu nivoa TAS (p = 0,228).

**Zaključak:**

Oksidativni stres i nivo HMBG-1 su povećani kod pacijenata sa wAMD-om, a pojačani oksidativni stres može biti povezan sa povećanom nekrozom tkiva i upalom. Stoga bi primenu antioksidativnog tretmana pored rutinske terapije trebalo razmotriti wAMD-u.

## Introduction

Age-related macular degeneration (AMD) is one of the most common causes of central vision loss in the elderly [1]. As a result of abnormalities in the Bruch's membrane, retinal pigment epithelium (RPE), photoreceptor, and choroid complex, AMD can often result in regional atrophy and/or neovascularization. For this reason, the disease is basically divided into two subgroups: wet and dry type [2].

AMD, is associated with various pathological factors such as chronic oxidative stress, decrease in autophagy, and chronic inflammation [3]. Although several local and systemic inflammatory molecules are recommended as markers for AMD, a specific and reliable marker has not been established. 

High Mobility Group Box-1 (HMGB-1), a nonhistone protein, is found in large amounts in the nucleus and plays a role in DNA transcription, replication, and repair [4]. HMGB-1 is a newly described inflammatory molecule that is a mediator of endotoxin shock and is elevated in the blood of septic patients [5]. The expression of HMGB-1 increases significantly in dry-type AMD and also increases the aging rate of RPE cells [6] [7]. In addition, the application of recombinant HMGB-1 initiates a pro-inflammatory response in human endothelial cells [5]. Considering the importance of these molecules and inflammatory status in AMD pathogenesis, it is understood that HMGB-1 is important in the prognosis and pathogenesis of this disease. However, apart from a few limited studies, which are all based on cell culture, there are no studies investigating the role and level of serum HMGB-1 in macular diseases in humans.

Recent research has provided a lot of evidence on the relationship between inflammation drusen and oxidative stress in the development of wet type AMD (wAMD). During inflammation, nitric oxide NO plays an important role and it may be released in high amounts with stimulation. Higher levels of NO in plasma have been reported in AMD [8]. NO is an important physiological regulator but it can form other reactive intermediates such as nitrite (NO_2_), peroxynitrite (ONOO), which can change the structure of tissues. Peroxynitrite changes the structure of molecules by adding a nitro group to the phenolic ring of tyrosine in proteins or in free form. Thus, 3-nitrotyrosine (3-NT) is a specific marker for oxidative damage to proteins and NO production [9]. In this case, 3-NT levels in AMD patients can provide information on pathogenesis and damage.

The formation and removal of free radicals in the living system occur in perfect balance, and the organism is not affected by these reactive molecules if this sensitive condition stabilizes the oxidative balance. If one of the steps to achieve this balance is interrupted, it causes oxidative stress that can lead to cell damage. Therefore, while oxidative stress is so important in AMD pathogenesis, plasma oxidant and antioxidant levels also gain importance. The total effects of all antioxidants in serum and other body fluids are measured according to the total antioxidant status (TAS), and the total effects of oxidants are determined by the total oxidant status (TOS). In addition, a global oxidative stress index (OSI) reflecting both oxidative and antioxidant responses is informative [10].

The relationship of AMD, whose inflammatory base has been demonstrated in many studies, with HMGB-1, an inflammation-related molecule, has not been previously examined and we aimed to contribute to the literature on this matter. We measured 3-NT levels which is a marker of nitrosative stress and hasn't studied in patients with AMD; and TOS, TAS, and OSI levels which is an indicator of oxidative stress that has been shown to be associated with AMD before, in serum of wAMD patients.

## Material and methods

### Study population

The study was approved by the Noninvasive Ethics Committee of Niğde Ömer Halisdemir University (The Decision Number: 2020/52) and written consent was obtained from each patient before taking the first blood samples. The study was conducted in accordance with the principles of the Helsinki Declaration. 30 patients with wAMD and 27 healthy controls constituted the study group of our research. All individuals included in the study groups were over 50 years old. Patients with other eye pathologies, such as glaucoma, uveitis, who had undergone intraocular surgery, smoking, and systemic disease were not included in the study. The diagnosis of wAMD was carried out by examination, fundus photographs, optical coherence tomography, and fundus fluorescein angiography. The control group of our study consisted of individuals without the systemic disease who had a complete healthy eyeexamination.

### Blood sample collection

Five mL of venous blood samples were taken into sterile biochemistry tubes with gel from the patient and control groups. Then serum samples were obtained by centrifuging at 1600xg for ten min. Sera samples were kept at -80°C until analysis day.

### Determination of Serum HMGB-1 and 3-Nitrotyrosine Levels

HMGB-1 and 3-Nitrotyrosine levels were measured with ELISA kits (Cusabio; CSB-E08223h and Yehua; YHB0033Hu-96, respectively) according to the instructions of the manufacturers.

### Measurement of TAS and TOS levels in the serum samples

TOS and TAS levels were measured using commercially available kits (Rel Assay, Mega Tıp, Gaziantep, Turkey) by Erel's colorimetric method. The results were expressed mmol Trolox equivalents/L for TAS; μmol H_2_O_2_ equiv/L for TOS [11] [12].

### Determination of oxidative stress index

OSI was calculated through the following formula:



\text{OSI} = [  \text{TOS} (\text{mmol Trolox equivalent}/\text{L})/\text{TAS}
(\mu \text{mol} \ \text{H}_2\text{O}_2 \ \text{equivalent} /\text{L})] \times 100

### Statistical analysis

Statistical analysis was made with IBM SPSS Statistics version 23 software. The normal distribution of the variables was assessed with the Shapiro-Wilks test. Statistical data were stated as mean±standard deviation (x̄ ± SD), or median and inter-quartile range for normally and non-normally distributed variables, respectively. Comparisons between groups for continuous variables were performed using the Student t-test (normal distribution) or the Mann-Whitney U test (non-normal distribution). Data were considered statistically significant at a value of p<0.05.

## Results

As can be seen in Table 1, there were 13 females and 17 males in the patient group while 10 females and 17 males in the control group. The mean age was 71.13 ± 9.37 years in the patient group, and 66.51±8.09 years in the controls. There was no significant difference between the groups in terms of gender (p=0.138) and age (p=0.053).

**Table 1:** Demographic data of patients and the control group SD, standard deviation;^ *^ p < 0.05

	Patient Group<br>(N=30)	Control Group<br>(N=27)	*p*
Mean Age<br>(year±SD)	71.13±9.37	66.51±8.09	0.053
Sex <br>(Female/Male)	13/17	10/17	0.138

Table 2 summarizes the statistical comparison of HMGB-1, 3-NT, TAS, TOS, and OSI levels determined in the sera samples of the study groups. Median levels of HMGB-1 were 137.51 pg/mL in wAMD patients and 95.93 pg/mL in controls. The median 3-NT levels were 951.53 nmol/L in the patient group and 900.33 nmol/L in the control group. The levels of HMGB-1 were meaningfully higher in the patient group than in the control group while there was no difference between the two groups in terms of 3-NT levels. Furtheremore, while there was no significant difference between TAS values, TOS and OSI levels were significantly higher in the patients then the control (Table 2). Lastly, we found a significant correlation coefficient between the OSI and HMGB-1 levels measured in the patient group (r^2^=0.6; p=0.0001).

**Table 2:** HMGB-1, 3-NT, TAS, TOS, and OSI levels inpatients with wAMD and the control group HMGB-1, TAS, TOS, and OSI levels were expressed as the median (min-max), whereas 3-NT levels as the mean ± standarddeviation. HMGB-1, High Mobility Group Box-1; 3-NT, 3-nitrotyrosine; TAS, Total antioxidant status; TOS, Total oxidant status; OSI, Oxidative stress index. ^* ^p < 0.05.

	Contor Group<br>(N=27)	Patient Group<br>(N=30)	*p*
HMGB-1<br>(pg/mL)	95.93<br>(92.90–114.63)	137.51<br>(126.47–166.17)	0.001*
3-NT (nmol/L)	900.33 ±197.47	951.53 ± 236.62	0.428
TAS (mmol<br>Trolox Equiv. /L)	1.73 (0.73–2.96)	2.00 (1.06–2.89)	0.228
TOS (μmol<br>H_2_O_2_ Equiv. /L)	7.93 (6.99–9.18)	7.03 (7.11–30.79)	0.001*
OSI (%)	0.56 (0.25–1.13)	0.79 (0.56–1.42)	0.045*

## Discussion

The retina is one of the tissues with the highest oxygen consumption in humans [13]. Indeed, Hanus et al. [14] reported that RPE cells undergo necrosis due to increased oxidative stress. As it is known, ROS attacks the cells, especially the lipids in the membrane, DNA, RNA, and proteins by disruptingtheir functions. Such a situation may increase the necrosis of RPE cells in wAMD patients. It has been suggested that RPE cell injury and death caused by oxidative stress promote the release of damage-associated molecular pattern molecules (DAMP) due to intracellular and extracellular damage. DAMP can stimulate immune and inflammatory responses and lead to AMD progression [15]. Consistent with these findings, we found that levels of TOS and OSI werehigher in wAMD patients in our study (p<0.05).

HMGB-1 is a large DAMP molecule that releases from the nucleus during necrosis and is secreted into the extracellular matrix and induces inflammation [16]. It has been reported that HMGB-1 is secreted from also necrotic RPE cells and induces inflammation [17]. HMGB-1 is a non-histone DNA binding protein that was first described by Goodwin and Johns in 1973 [18]. It plays an important role in DNA transcription, replication, and repair also contributes to the collection of nuclear proteins. In the cytoplasm, it functions as a signal regulator and takes a role in the inflammatory cascade, and acts as a pro-inflammatory cytokine in the extracellular environment [19]. HMGB-1 serum levels, which have an important role in mediating inflammation, have been shown to be high in various diseases [20]. Furthermore, HMGB1plays an important regulatory role in angiogenesis and it affects many angiogenesis-related conditions such as proliferative diabetic retinopathy, cancer, and wound healing via p53. Thus, HMGB1 is a promising therapeutic target in many malignancies such as prostate and colon cancer, and epidermal tumors [21] [22] [23] [24].

HMGB-1, which is intertwined with inflammation and oxidative stress, also attracts attention in the field of ophthalmology. Sakamoto et al. [25] showed that HMGB-1 induced cell death of retinal ganglion cells with increased oxidative stress in the rat retina. Chang et al, [26] reported that the release of HMGB-1 peptides caused by hypoxia-regulated the production of angiofibrogenic factors in RPE cells, thereby contributing to the pathogenesis of hypoxiaassociated diabetic retinopathy. Watanabe et al. [27] reported that the extracellular HMGB-1 contributes to ocular inflammation in autoimmune uveoretinitis. Murakami et al. [28] investigated the vitreous of retinitis pigmentosa (RP) patients and found a significant release of extracellular HMGB-1 associated with necrotic cell death and they reported that HMGB-1 could be a new therapeutic target in RP. In a study on pluripotent stem cells from healthy individuals, Sun et al. [7] showed that HMGB-1 caused RPE cell aging. They also reported that HMGB-1 upregulated Caveolin-1, which is also associated with RPE cell aging. With these results, they demonstrated that HMGB-1 may be associated with RPE cell aging and may play a role in AMD pathogenesis, thus suggesting that it is a potential therapeutic target to prevent the progression of RPE cell aging. We found significantly higher HMGB-1 levels in wAMD patient sera (137.51 pg/mL) compared to the control group (95.93 pg/mL) in our study and to our knowledge this was the first study in the literature to examine the HMGB-1 level in the serum of wAMD patients (p=0.001).

Nitrotyrosine is an indicator of the production of reactive nitrogen species and this end product causes damage to cellular components. Therefore, tyrosine nitration has been recently investigated in the pathogenesis of diseases [29]. In a study by Thomson [30], 3-nitrotyrosine levels were found to be associated with higher cardiovascular risk. Bandookwala et al. [31] reported that the change in the nitrotyrosine profile occurred in the presymptomatic stage of the neurodegenerative diseases and this could be used for early diagnosis. Qian et al. [32] found that nitrotyrosine level was associated with mortality in patients with acute kidney injury. Khan et al. [33] reported that serum 3-NT has increased in SLE patients and preventing nitrosative damage could be new treatment methods for some diseases. Nitrosative stress also attracts attention in the field of ophthalmology. It was shown that the formation of nitrotyrosine significantly increases in the diabetic retina and is an inflammatory element in the development of diabetic retinopathy [29]. Wang et al. [34] reported that 3-nitrotyrosine levels in blood serum were higher in type 2 diabetic patients than in healthy individuals. In RP, nitrosative stress has been reported to be effective in cone photoreceptor deaths [35]. In another study, it has been reported that retinal nitrosative stress, plays an important role in retinal ganglion cell loss in glaucoma [36]. In a study in the tissue culture, it was reported that the nonenzymatic nitration of the RPE basement membrane may have significant harmful effects on the RPE function and they found that the accumulation of 3nitrotyrosine increased with the age of the patient on the human Bruch membrane [8]. Wang et al. [37] reported that nitrite-induced changes in the normal basement membrane can mimic the harmful effects of the aging Bruch membrane on RPE function. In line with these findings, in our study, in which we planned to evaluate the importance of 3-nitrotyrosine in AMD pathogenesis, we found that the level of serum 3-nitrotyrosine did not show a statistically significant difference between the two groups (p=0.428). Although our findings indicate that nitrosative stress is not significantly effective in patients with AMD, different results can be obtained in large-scale studies conducted with study groups consisting of more individuals, thus we consider that additional studies are needed.

Free radicals are reactive molecules and these molecules damage cell components, and oxidative stress occurs [38]. The formation and removal of free radicals in the body occur in perfect balance, imbalance between these two processes causes oxidative stress that can cause cell damage in the living systems. Oxidative stress plays an important role in the pathogenesis of many diseases [39]. Icel et al. [40] reported that TOS level was found to be significantly higher while TAS was found to be low in the study of diabetic rats. Oruç et al. [41] found that the TOS value was high while the TAS value was found low in humorous aqueous of pseudoexfoliation patients, and they reported that normal these levels may slow the progression of pseudoexfoliation syndrome. In a study conducted by Altını ık et al. [42] in patients with retinal vein occlusion, it was found that the oxidative stress index in humor aqueous was high, while TAS values were low. Recently, the relationship between AMD and oxidative stress has drawn attention in a study [13] [14]. Elbay et al. [43] investigated wAMD patients and reported that serum TOS levels increased and TAS values decreased in wAMD patients. In the literature, there is no other study examining TOS and TAS values in wAMD patients. In our study, it was found that TOS levels increased and there is no difference between TAS values. We believe that our study is the second study on TOS and TAS values in AMD patients. As a result of high TOS values, OSI was found significantly higher in the patient group.

## Conclusion

We have found significantly higher OSI in wAMD patients and thus oxidative stress may have important roles in AMD pathogenesis. The high TOS and normal TAS values that we have found indicate increased oxidative stress and a sufficient antioxidant system in wAMD patients. Furthermore, increased HMGB-1 levels in patients with wAMD and determined the significant correlation between OSI and this parameter may suggest that increased tissue necrosis and inflammation in these patients are associated with OSI. However, further studies are needed to examine the effects of OSI and inflammation on the pathogenesis of wAMD and their relationship in this disease.

## Dodatak

### Authors’ contributions

All authors have read the final manuscript within their respective areas of expertise and participated sufficiently in the study to take responsibility for it and accept its conclusions.

### Declarations

Funding: Not applicable.

### Research involving Human Participants and/or Animals

This article does not contain any studies with human participants or animals performed by any of the authors.

### Conflict of interest statement

All the authors declare that they have no conflict of interest in this work.
